# (*E*)-1-(2-Phenyl­diazen-2-ium-1-yl)naph­thalen-2-olate

**DOI:** 10.1107/S1600536813020102

**Published:** 2013-07-27

**Authors:** Hassiba Bougueria, Souheyla Chetioui, Issam Boudraa, Abd el kader Bouchoul, Salah Eddine Bouaoud

**Affiliations:** a Unité de Recherche de Chimie de l’Environnement et Moléculaire Structurale (CHEMS), Département de Chimie, Université Mentouri de Constantine 1, 25000 Constantine, Algeria

## Abstract

In the title zwitterionic compound, C_16_H_12_N_2_O, the dihedral angle between the phenyl ring and the naphthalene ring system is 17.85 (8)°; an intra­molecular N—H⋯O hydrogen bond occurs. In the crystal, π–π stacking is observed between naphthalene ring systems of adjacent mol­ecules, the centroid–centroid distance being 3.6486 (11) Å.

## Related literature

For general background to azo compounds and their applications in the fields of dyes, pigments and advanced materials, see: Biswas & Umapathy (2000 ▶); Willner & Rubin (1996 ▶); Hunger (2003 ▶); Catino & Farris (1985 ▶); Zollinger (2003 ▶); Bahatti & Seshadri (2004 ▶); Taniike *et al.* (1996 ▶); Fadda *et al.* (1994 ▶); Bach *et al.* (1996 ▶); Clark & Hester (1993 ▶). For the synthesis, see: Wang *et al.* (2003 ▶).
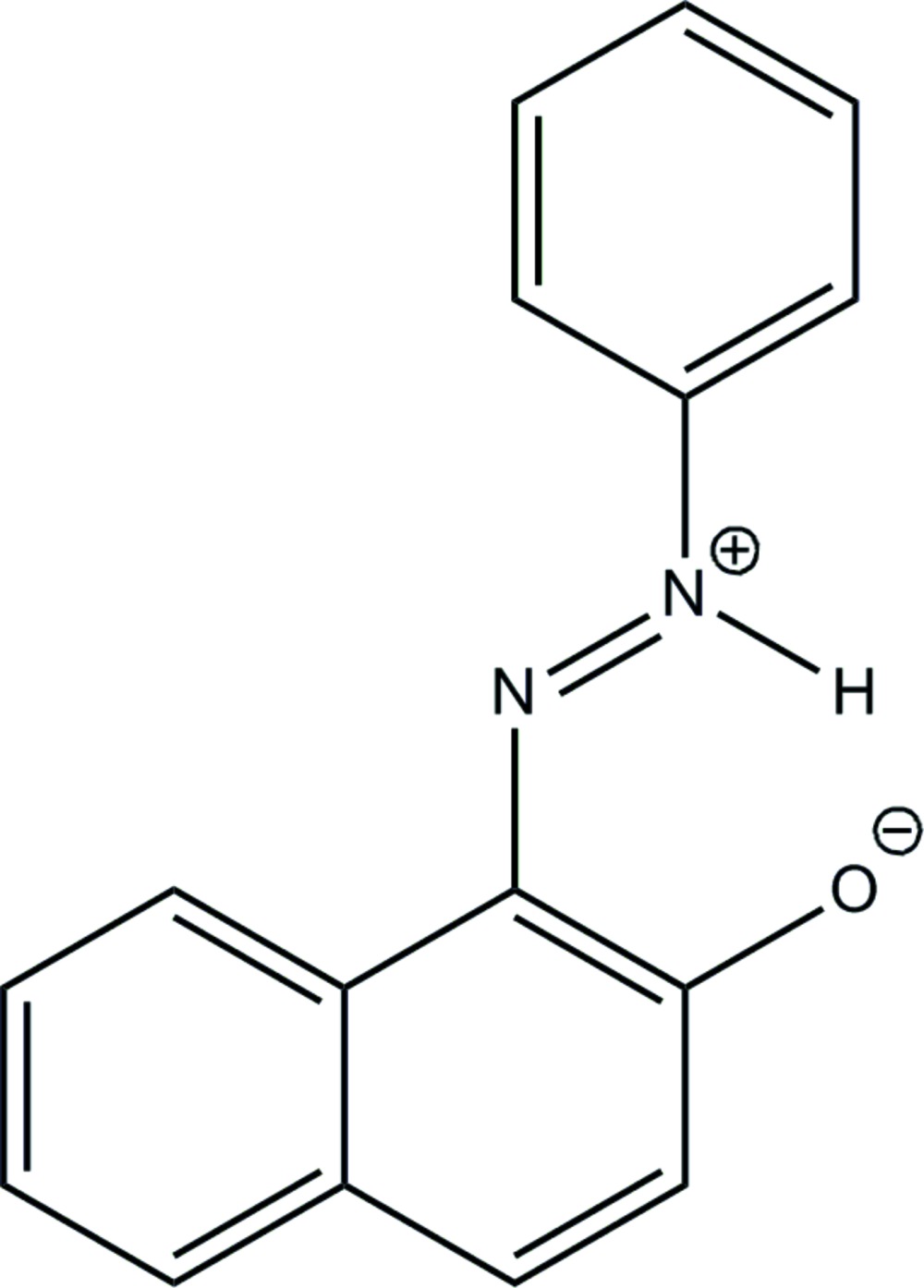


## Experimental

### 

#### Crystal data

C_16_H_12_N_2_O
*M* *_r_* = 248.28
Monoclinic, 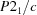

*a* = 13.0800 (12) Å
*b* = 13.5170 (13) Å
*c* = 7.0080 (4) Å
β = 94.140 (6)°
*V* = 1235.80 (18) Å^3^
*Z* = 4
Mo *K*α radiation
μ = 0.09 mm^−1^
*T* = 150 K
0.26 × 0.22 × 0.17 mm

#### Data collection

Nonius KappaCCD diffractometer
4092 measured reflections
2139 independent reflections
1546 reflections with *I* > 2σ(*I*)
*R*_int_ = 0.026

#### Refinement

*R*[*F* ^2^ > 2σ(*F* ^2^)] = 0.054
*wR*(*F* ^2^) = 0.144
*S* = 1.06
2139 reflections
172 parameters
H-atom parameters constrained
Δρ_max_ = 0.18 e Å^−3^
Δρ_min_ = −0.19 e Å^−3^

### 

Data collection: *KappaCCD Server Software* (Nonius, 1999 ▶); cell refinement: *KappaCCD Server Software*; data reduction: *DENZO* and *SCALEPACK* (Otwinowski & Minor, 1997 ▶); program(s) used to solve structure: *SHELXS86* (Sheldrick, 2008 ▶); program(s) used to refine structure: *SHELXL97* (Sheldrick, 2008 ▶); molecular graphics: *ORTEP-3 for Windows* (Farrugia, 2012 ▶); software used to prepare material for publication: *WinGX* (Farrugia, 2012 ▶).

## Supplementary Material

Crystal structure: contains datablock(s) global, I. DOI: 10.1107/S1600536813020102/xu5718sup1.cif

Structure factors: contains datablock(s) I. DOI: 10.1107/S1600536813020102/xu5718Isup2.hkl

Supplementary material file. DOI: 10.1107/S1600536813020102/xu5718Isup3.cml

Additional supplementary materials:  crystallographic information; 3D view; checkCIF report

## Figures and Tables

**Table 1:** Hydrogen-bond geometry (Å, °)

*D*—H⋯*A*	*D*—H	H⋯*A*	*D*⋯*A*	*D*—H⋯*A*
N1—H1⋯O1	0.94	1.73	2.5346 (19)	141

**Fig. 1.**
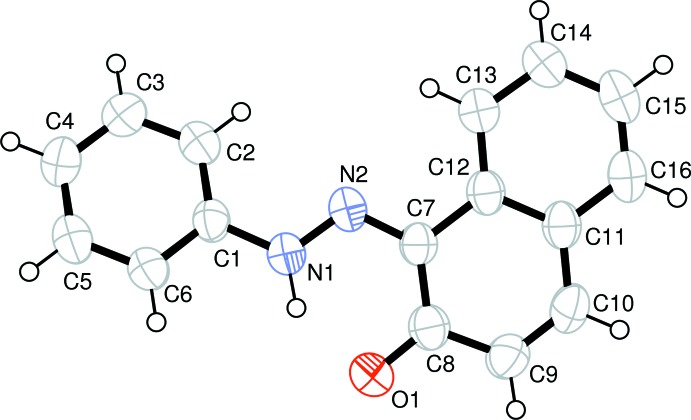
View of the molecular structure of the title compound, with atom labelling. Displacement ellipsoids are drawn at the 50% probability level.

## supplementary crystallographic information

### Comment

Azo compounds are very important in the field of dyes, pigments and advanced materials (Hunger, 2003). It has been known for many years that the azo compounds are the most widely used class of dyes, due to their versatile applications in various fields such as the dyeing of textile fibers, the coloring of different materials, colored plastics and polymers, biological-medical studies and advanced applications in organic syntheses (Catino & Farris, 1985; Zollinger, 2003; Bahatti & Seshadri, 2004; Taniike *et al.*, 1996; Fadda *et al.*, 1994). They are also used in the fields of nonlinear optics and optical data storage (Taniike *et al.*, 1996; Bach *et al.*, 1996; Clark & Hester, 1993). Their optical properties depend on not only the spectroscopic properties of the molecules but also their crystallographic arrangements (Biswas & Umapathy, 2000; Willner & Rubin, 1996).

We report here in the crystal structure of the title compound, obtained through the diazotization of aniline followed by a coupling reaction with 2-naphthol.

The molecule of the title compound, with the atom numbering scheme, is shown in Fig. 1, crystallizes in the monoclinic space group *P*21/*c.*

The molecular structure of C_16_ H_12_ N_2_ O is illustrated in Fig. 1. The molecule adopts an anti–configuration with the two aryl groups reside on the opposite side of azo–group. The dihedral angle between the benzene ring and naphthalene ring is 17.85 (8)°. An intramolecular N—H···O hydrogen bond is found (Table 1). It is more interesting, that hydrogen atom in the OH-group has transfer to N atom in the azo-group to form the structure of dipolar ion. Moreover, different Fourier map indicate hydrogen site location is closer to nitrogen atom of azo-group. In the crystal molecules are packed by the weak π–π interactions with the closest approach between centroids of aromatic rings is 3.6486 (11) Å.

### Experimental

The compound was synthesized according to the literature procedure of other aromatic azo-compounds (Wang *et al.*, 2003). Red prism of the compound were obtained by slow evaporation at room temperature from an aqueous solution containing water/THF (1/1, v/v).

### Refinement

N-bound H atom was located in a differece Fourier map and refined with N—H distance constraint of 0.94 Å, other H atoms were placed in calculated positions and refined in riding mode. U_iso_(H) = 1.2U_eq_(N,C).

### Crystal data

**Table:**

C_16_H_12_N_2_O	*F*(000) = 520
*M**_r_* = 248.28	*D*_x_ = 1.334 Mg m^−^^3^
Monoclinic, *P*2_1_/*c*	Mo *K*α radiation, λ = 0.71073 Å
Hall symbol: -P 2ybc	Cell parameters from 2200 reflections
*a* = 13.0800 (12) Å	θ = 1.0–25.4°
*b* = 13.5170 (13) Å	µ = 0.09 mm^−^^1^
*c* = 7.0080 (4) Å	*T* = 150 K
β = 94.140 (6)°	Prism, red
*V* = 1235.80 (18) Å^3^	0.26 × 0.22 × 0.17 mm
*Z* = 4	

### Data collection

**Table:**

Nonius KappaCCD diffractometer	1546 reflections with *I* > 2σ(*I*)
Radiation source: fine-focus sealed tube	*R*_int_ = 0.026
Graphite monochromator	θ_max_ = 25.4°, θ_min_ = 2.2°
Detector resolution: 9 pixels mm^-1^	*h* = −15→15
CCD scans	*k* = −16→16
4092 measured reflections	*l* = −7→7
2139 independent reflections	

### Refinement

**Table:**

Refinement on *F*^2^	Primary atom site location: structure-invariant direct methods
Least-squares matrix: full	Secondary atom site location: difference Fourier map
*R*[*F*^2^ > 2σ(*F*^2^)] = 0.054	Hydrogen site location: inferred from neighbouring sites
*wR*(*F*^2^) = 0.144	H-atom parameters constrained
*S* = 1.06	*w* = 1/[σ^2^(*F*_o_^2^) + (0.0756*P*)^2^ + 0.113*P*] where *P* = (*F*_o_^2^ + 2*F*_c_^2^)/3
2139 reflections	(Δ/σ)_max_ = 0.001
172 parameters	Δρ_max_ = 0.18 e Å^−^^3^
0 restraints	Δρ_min_ = −0.19 e Å^−^^3^

### Special details

**Table:**

Geometry. All esds (except the esd in the dihedral angle between two l.s. planes) are estimated using the full covariance matrix. The cell esds are taken into account individually in the estimation of esds in distances, angles and torsion angles; correlations between esds in cell parameters are only used when they are defined by crystal symmetry. An approximate (isotropic) treatment of cell esds is used for estimating esds involving l.s. planes.
Refinement. Refinement on F^2^ for ALL reflections except those flagged by the user for potential systematic errors. Weighted R-factors wR and all goodnesses of fit S are based on F^2^, conventional R-factors R are based on F, with F set to zero for negative F^2^. The observed criterion of F^2^ > 2sigma(F^2^) is used only for calculating -R-factor-obs etc. and is not relevant to the choice of reflections for refinement. R-factors based on F^2^ are statistically about twice as large as those based on F, and R-factors based on ALL data will be even larger.

### Fractional atomic coordinates and isotropic or equivalent isotropic displacement parameters (Å^2^)

**Table:**

	*x*	*y*	*z*	*U*_iso_*/*U*_eq_	
N1	0.20061 (11)	0.46423 (10)	0.4042 (2)	0.0542 (4)	
H1	0.1287	0.4666	0.4047	0.065*	
N2	0.23990 (12)	0.37596 (10)	0.4036 (2)	0.0524 (4)	
O1	0.02112 (10)	0.39323 (10)	0.3599 (2)	0.0688 (4)	
C1	0.26520 (14)	0.54729 (12)	0.4226 (2)	0.0510 (4)	
C2	0.36888 (15)	0.54252 (15)	0.3965 (3)	0.0630 (5)	
H2	0.3987	0.4832	0.3627	0.076*	
C3	0.42736 (16)	0.62723 (15)	0.4215 (3)	0.0700 (6)	
H3	0.4972	0.6246	0.4052	0.084*	
C4	0.38374 (17)	0.71530 (16)	0.4701 (3)	0.0727 (6)	
H4	0.4240	0.7718	0.4868	0.087*	
C5	0.28076 (17)	0.71967 (15)	0.4938 (3)	0.0718 (6)	
H5	0.2511	0.7792	0.5267	0.086*	
C6	0.22090 (15)	0.63589 (13)	0.4690 (3)	0.0603 (5)	
H6	0.1509	0.6392	0.4836	0.072*	
C7	0.17482 (13)	0.29843 (12)	0.3881 (2)	0.0490 (4)	
C8	0.06510 (15)	0.30859 (14)	0.3645 (3)	0.0563 (5)	
C9	0.00491 (15)	0.22000 (16)	0.3455 (3)	0.0651 (5)	
H9	−0.0660	0.2249	0.3272	0.078*	
C10	0.04871 (16)	0.12996 (15)	0.3537 (3)	0.0634 (5)	
H10	0.0069	0.0744	0.3419	0.076*	
C11	0.15749 (15)	0.11656 (13)	0.3798 (2)	0.0543 (5)	
C12	0.22131 (14)	0.20077 (12)	0.3962 (2)	0.0509 (4)	
C13	0.32739 (15)	0.18637 (15)	0.4207 (3)	0.0635 (5)	
H13	0.3709	0.2408	0.4328	0.076*	
C14	0.36788 (17)	0.09244 (15)	0.4270 (3)	0.0728 (6)	
H14	0.4385	0.0840	0.4437	0.087*	
C15	0.30467 (18)	0.01006 (16)	0.4088 (3)	0.0723 (6)	
H15	0.3329	−0.0531	0.4119	0.087*	
C16	0.20132 (17)	0.02215 (14)	0.3865 (3)	0.0647 (5)	
H16	0.1591	−0.0332	0.3755	0.078*	

### Atomic displacement parameters (Å^2^)

**Table:**

	*U*^11^	*U*^22^	*U*^33^	*U*^12^	*U*^13^	*U*^23^
N1	0.0545 (9)	0.0442 (9)	0.0639 (10)	0.0000 (7)	0.0040 (7)	0.0008 (6)
N2	0.0613 (9)	0.0424 (9)	0.0534 (9)	0.0008 (7)	0.0040 (6)	−0.0001 (6)
O1	0.0601 (8)	0.0556 (9)	0.0902 (10)	0.0066 (7)	0.0019 (7)	−0.0011 (7)
C1	0.0580 (10)	0.0412 (10)	0.0534 (10)	−0.0017 (8)	0.0010 (8)	0.0031 (7)
C2	0.0625 (12)	0.0510 (12)	0.0760 (13)	0.0055 (9)	0.0072 (9)	−0.0013 (9)
C3	0.0567 (11)	0.0630 (14)	0.0899 (15)	−0.0042 (10)	0.0027 (10)	0.0022 (10)
C4	0.0723 (14)	0.0514 (12)	0.0939 (16)	−0.0106 (11)	0.0019 (11)	−0.0008 (10)
C5	0.0777 (14)	0.0455 (11)	0.0925 (16)	0.0029 (10)	0.0086 (11)	−0.0029 (10)
C6	0.0580 (11)	0.0488 (11)	0.0743 (13)	0.0035 (9)	0.0071 (9)	0.0036 (9)
C7	0.0558 (10)	0.0446 (10)	0.0466 (10)	−0.0025 (8)	0.0039 (7)	−0.0027 (7)
C8	0.0641 (12)	0.0525 (11)	0.0523 (11)	−0.0002 (9)	0.0044 (8)	−0.0025 (8)
C9	0.0586 (11)	0.0626 (13)	0.0739 (13)	−0.0071 (10)	0.0031 (9)	−0.0106 (9)
C10	0.0699 (13)	0.0528 (12)	0.0679 (12)	−0.0153 (10)	0.0065 (9)	−0.0085 (9)
C11	0.0702 (12)	0.0464 (11)	0.0467 (10)	−0.0040 (9)	0.0061 (8)	−0.0048 (7)
C12	0.0628 (11)	0.0451 (10)	0.0450 (10)	−0.0017 (8)	0.0059 (7)	−0.0012 (7)
C13	0.0623 (12)	0.0501 (12)	0.0780 (13)	−0.0001 (9)	0.0039 (10)	0.0015 (9)
C14	0.0701 (13)	0.0554 (13)	0.0928 (16)	0.0097 (11)	0.0050 (11)	0.0043 (11)
C15	0.0933 (16)	0.0450 (12)	0.0788 (14)	0.0100 (11)	0.0081 (11)	0.0016 (9)
C16	0.0843 (14)	0.0447 (11)	0.0654 (12)	−0.0052 (10)	0.0079 (10)	−0.0032 (8)

### Geometric parameters (Å, º)

**Table:**

N1—N2	1.2993 (19)	C7—C12	1.453 (2)
N1—C1	1.405 (2)	C8—C9	1.434 (3)
N1—H1	0.9418	C9—C10	1.345 (3)
N2—C7	1.350 (2)	C9—H9	0.9300
O1—C8	1.280 (2)	C10—C11	1.433 (3)
C1—C6	1.380 (2)	C10—H10	0.9300
C1—C2	1.383 (3)	C11—C16	1.398 (3)
C2—C3	1.381 (3)	C11—C12	1.411 (2)
C2—H2	0.9300	C12—C13	1.399 (3)
C3—C4	1.374 (3)	C13—C14	1.375 (3)
C3—H3	0.9300	C13—H13	0.9300
C4—C5	1.370 (3)	C14—C15	1.387 (3)
C4—H4	0.9300	C14—H14	0.9300
C5—C6	1.381 (3)	C15—C16	1.359 (3)
C5—H5	0.9300	C15—H15	0.9300
C6—H6	0.9300	C16—H16	0.9300
C7—C8	1.439 (3)		
			
N2—N1—C1	119.91 (15)	O1—C8—C7	122.06 (16)
N2—N1—H1	115.2	C9—C8—C7	117.85 (17)
C1—N1—H1	124.6	C10—C9—C8	121.51 (18)
N1—N2—C7	117.78 (15)	C10—C9—H9	119.2
C6—C1—C2	120.28 (17)	C8—C9—H9	119.2
C6—C1—N1	117.08 (16)	C9—C10—C11	122.41 (18)
C2—C1—N1	122.64 (16)	C9—C10—H10	118.8
C3—C2—C1	118.96 (18)	C11—C10—H10	118.8
C3—C2—H2	120.5	C16—C11—C12	119.65 (18)
C1—C2—H2	120.5	C16—C11—C10	121.37 (17)
C4—C3—C2	120.9 (2)	C12—C11—C10	118.98 (16)
C4—C3—H3	119.5	C13—C12—C11	118.24 (16)
C2—C3—H3	119.5	C13—C12—C7	122.67 (16)
C5—C4—C3	119.80 (19)	C11—C12—C7	119.09 (17)
C5—C4—H4	120.1	C14—C13—C12	120.57 (18)
C3—C4—H4	120.1	C14—C13—H13	119.7
C4—C5—C6	120.19 (19)	C12—C13—H13	119.7
C4—C5—H5	119.9	C13—C14—C15	120.8 (2)
C6—C5—H5	119.9	C13—C14—H14	119.6
C1—C6—C5	119.85 (18)	C15—C14—H14	119.6
C1—C6—H6	120.1	C16—C15—C14	119.7 (2)
C5—C6—H6	120.1	C16—C15—H15	120.2
N2—C7—C8	123.58 (16)	C14—C15—H15	120.2
N2—C7—C12	116.27 (16)	C15—C16—C11	121.02 (19)
C8—C7—C12	120.16 (16)	C15—C16—H16	119.5
O1—C8—C9	120.09 (18)	C11—C16—H16	119.5
			
C1—N1—N2—C7	179.15 (14)	C8—C9—C10—C11	−0.6 (3)
N2—N1—C1—C6	−164.58 (16)	C9—C10—C11—C16	−179.56 (17)
N2—N1—C1—C2	15.3 (2)	C9—C10—C11—C12	−0.5 (3)
C6—C1—C2—C3	1.3 (3)	C16—C11—C12—C13	−0.6 (2)
N1—C1—C2—C3	−178.58 (17)	C10—C11—C12—C13	−179.67 (16)
C1—C2—C3—C4	−0.5 (3)	C16—C11—C12—C7	179.69 (14)
C2—C3—C4—C5	−0.2 (3)	C10—C11—C12—C7	0.6 (2)
C3—C4—C5—C6	0.0 (3)	N2—C7—C12—C13	0.7 (2)
C2—C1—C6—C5	−1.5 (3)	C8—C7—C12—C13	−179.37 (16)
N1—C1—C6—C5	178.41 (17)	N2—C7—C12—C11	−179.68 (14)
C4—C5—C6—C1	0.8 (3)	C8—C7—C12—C11	0.3 (2)
N1—N2—C7—C8	2.6 (2)	C11—C12—C13—C14	0.5 (3)
N1—N2—C7—C12	−177.42 (13)	C7—C12—C13—C14	−179.82 (17)
N2—C7—C8—O1	−1.2 (3)	C12—C13—C14—C15	0.1 (3)
C12—C7—C8—O1	178.80 (15)	C13—C14—C15—C16	−0.7 (3)
N2—C7—C8—C9	178.63 (15)	C14—C15—C16—C11	0.6 (3)
C12—C7—C8—C9	−1.3 (2)	C12—C11—C16—C15	0.1 (3)
O1—C8—C9—C10	−178.64 (17)	C10—C11—C16—C15	179.11 (18)
C7—C8—C9—C10	1.5 (3)		

### Hydrogen-bond geometry (Å, º)

**Table:**

*D*—H···*A*	*D*—H	H···*A*	*D*···*A*	*D*—H···*A*
N1—H1···O1	0.94	1.73	2.5346 (19)	141

## References

[bb1] Bach, H., Anderle, K., Fuhrmann, Th. & Wendorff, J. H. (1996). *J. Phys. Chem.* **100**, 4135–4140.

[bb2] Bahatti, H. S. & Seshadri, S. (2004). *Color. Technol.* **120**, 151–155.

[bb3] Biswas, N. & Umapathy, S. (2000). *J. Phys. Chem. A*, **104**, 2734–2745.

[bb4] Catino, S. C. & Farris, R. E. (1985). *Azo dyes, in Concise Encyclopedia of Chemical Technology*, edited by M. Grayson, pp. 142–144. New York: John Wiley and Sons.

[bb5] Clark, R. J. H. & Hester, R. E. (1993). *Spectroscopy of New Materials: Advances in Spectroscopy*, edited by R. J. H. Clark & R. E. Hester. New York: John Wiley and Sons.

[bb6] Fadda, A. A., Etmen, H. A., Amer, F. A., Barghout, M. & Mohammed, K. S. J. (1994). *J. Chem. Technol. Biotechnol.* **61**, 343–349.

[bb7] Farrugia, L. J. (2012). *J. Appl. Cryst.* **45**, 849–854.

[bb8] Hunger, K. (2003). *Industrial Dyes, Chemistry, Properties and Applications*, edited by K. Hunger, pp. 20–35. Weinheim: Wiley-VCH.

[bb9] Nonius (1999). *KappaCCD Server Software* Nonius BV, Delft, The Nertherlands.

[bb10] Otwinowski, Z. & Minor, W. (1997). *Methods in Enzymology*, Vol. 276, *Macromolecular Crystallography*, Part A, edited by C. W. Carter Jr & R. M. Sweet, pp. 307–326. New York: Academic Press.

[bb11] Sheldrick, G. M. (2008). *Acta Cryst.* A**64**, 112–122. 10.1107/S0108767307043930 18156677

[bb12] Taniike, K., Matsumoto, T., Sato, T., Ozaki, Y., Nakashima, K. & Iriyama, K. (1996). *J. Phys. Chem.* **100**, 15508–15516.

[bb13] Wang, M., Funabiki, K. & Matsui, M. (2003). *Dyes Pigments*, **57**, 77–86.

[bb14] Willner, I. & Rubin, S. (1996). *Angew. Chem. Int. Ed. Engl.* **35**, 367–385.

[bb15] Zollinger, H. (2003). *Colour Chemistry: Synthesis, Properties and Applications of Organic Dyes and Pigments*, edited by H. Zollinger, 3rd rev. ed. Weinheim: Wiley-VCH.

